# Cost-effectiveness of trans-abdominal ultrasound for gallbladder cancer surveillance in patients with gallbladder polyps less than 10 mm in the United Kingdom

**DOI:** 10.1093/bjr/tqaf024

**Published:** 2025-02-05

**Authors:** Julia Lowin, Bernadette Sewell, Matthew Prettyjohns, Angela Farr, Kieran G Foley

**Affiliations:** Swansea Centre for Health Economics (SCHE), Swansea University, Swansea SA2 8PP, United Kingdom; Swansea Centre for Health Economics (SCHE), Swansea University, Swansea SA2 8PP, United Kingdom; Health Technology Wales, Cardiff CF10 4PL, United Kingdom; Swansea Centre for Health Economics (SCHE), Swansea University, Swansea SA2 8PP, United Kingdom; Division of Cancer & Genetics, School of Medicine, Cardiff University, Cardiff CF14 4XN, United Kingdom

**Keywords:** gallbladder polyp, gallbladder cancer, ultrasound, cost-effectiveness

## Abstract

**Objectives:**

Gallbladder polyps (GBPs) are commonly detected with trans-abdominal ultrasound (TAUS). Gallbladder cancer (GBC) is associated with GBPs but the risk of malignancy is low. International guidelines recommend ultrasound surveillance (USS) in selected cases of GBPs <10 mm, with cholecystectomy advised if the polyp size increases. USS (including potential cholecystectomies) is resource intense. We evaluated the costs and potential cost-effectiveness of USS in a theoretical UK patient cohort with GBPs.

**Methods:**

A health economic model mapped expected management pathways over 2 years for 1000 GBP patients with and without USS, stratified by the initial size of GBP (<6 mm and 6–9 mm). We estimated USS resource and costs under alternate referral thresholds for cholecystectomy. Clinical data were extracted from a large-scale cohort study. TAUS and cholecystectomy costs were based on NHS tariffs. GBC costs were estimated from the literature. Outcomes included USS costs, expected numbers of GBC, and incremental cost for each case of GBC avoided.

**Results:**

The 2-year additional cohort costs of USS (*n* = number of cholecystectomies) were estimated between £213 441 (*n* = 50) and £750 045 (*n* = 253) in GBPs <6 mm and between £420 275 (*n* = 165) and £531 297 (*n* = 207) in GBPs 6–9 mm, balanced against avoidance of 1.3 (<6 mm) and 8.7 (6–9 mm) cases of GBC. Model findings were robust to plausible changes in inputs.

**Conclusions:**

Using published data, we demonstrated that, in patients with GBPs <10 mm, the costs of USS to avoid GBC outweigh potential GBC cost offsets and would result in high rates of cholecystectomy. Additional evidence is needed to establish the formal cost-effectiveness of GBP USS in the UK.

**Advances in knowledge:**

• We developed a health economic model, based on published data, to evaluate the cost-effectiveness of guideline-recommended ultrasound surveillance (USS) in patients with gallbladder polyps measuring less than 10 mm in the UK.

• The analysis provides a transparent platform to explore potential numbers of trans-abdominal ultrasound studies and cholecystectomies that might be expected if USS protocols are adhered to and discovers important gaps in current evidence that could be filled by additional targeted research.

## Introduction

Gallbladder polyps (GBPs) occur in approximately 2%–3% of the general adult population and are often detected incidentally, without symptoms, following trans-abdominal ultrasound (TAUS). GBPs have historically been linked to the subsequent development of gallbladder cancer (GBC), and guidelines recommend removal of the gallbladder (cholecystectomy) for patients presenting with GBPs measuring 10 mm or more, and mid- to long-term ultrasound surveillance (USS) for patients with GBPs less than 10 mm (limited to 2 years for stable GBPs).^1^^,^^2^ USS identifies GBPs that change substantially in size between scans which is thought to be associated with underlying malignancy. GBC often presents at an advanced stage and is consequently incurable, so early detection and cholecystectomy is an established management strategy.^2^ However, GBC is rare, affecting less than 0.001% of the general population, with less than 1% of GBP patients expected to develop GBC.^2^^,^^3^

Management of rare disease through large-scale surveillance is challenging,^4^^,^^5^ and the USS and subsequent surgical follow-up of GBPs less than 10 mm contributes substantially to radiology and surgical resource use.^6^ Recent updates to European radiology guidelines removed the recommendation for GBP follow-up less than 6 mm (in the absence of additional risk factors) but continue to recommend follow-up of GBPs presenting at 6–9 mm.^2^ However, the benefits of follow-up in a UK NHS setting are not well defined, and guidelines may not reflect the singularities of the UK NHS diagnostic landscape. Current papers, including the 2022 consensus statement from the US Society of Radiologists in Ultrasound,^7^^,^^8^ challenge the usefulness of USS for GBPs, questioning a direct clinical link between GBP and GBC, and highlighting the consistency in rates of GBC despite the increased rates of TAUS and cholecystectomy.^9^

Health economics provides a formal framework to assess the balance of costs and benefits associated with a given intervention through estimation of its relative cost-effectiveness. Recent UK studies based on projections of GBP pathways in individual hospital settings predict cost savings when the costs of USS schemes are compared against the costs of potential future GBCs avoided through delivery of the scheme.^10^^,^^11^ However, it is unclear whether the clinical and cost findings would be generalisable or realisable across other UK settings.^2^ Based on current high cost and resource constraints within the UK NHS, further exploration of the potential costs and cost-effectiveness of USS for GBPs measuring less than 10 mm in size is warranted. This health economic study evaluated routine TAUS follow-up management compared to a no follow-up strategy, to investigate the cost-effectiveness of USS in this patient group.

## Methods

**Figure 1.**
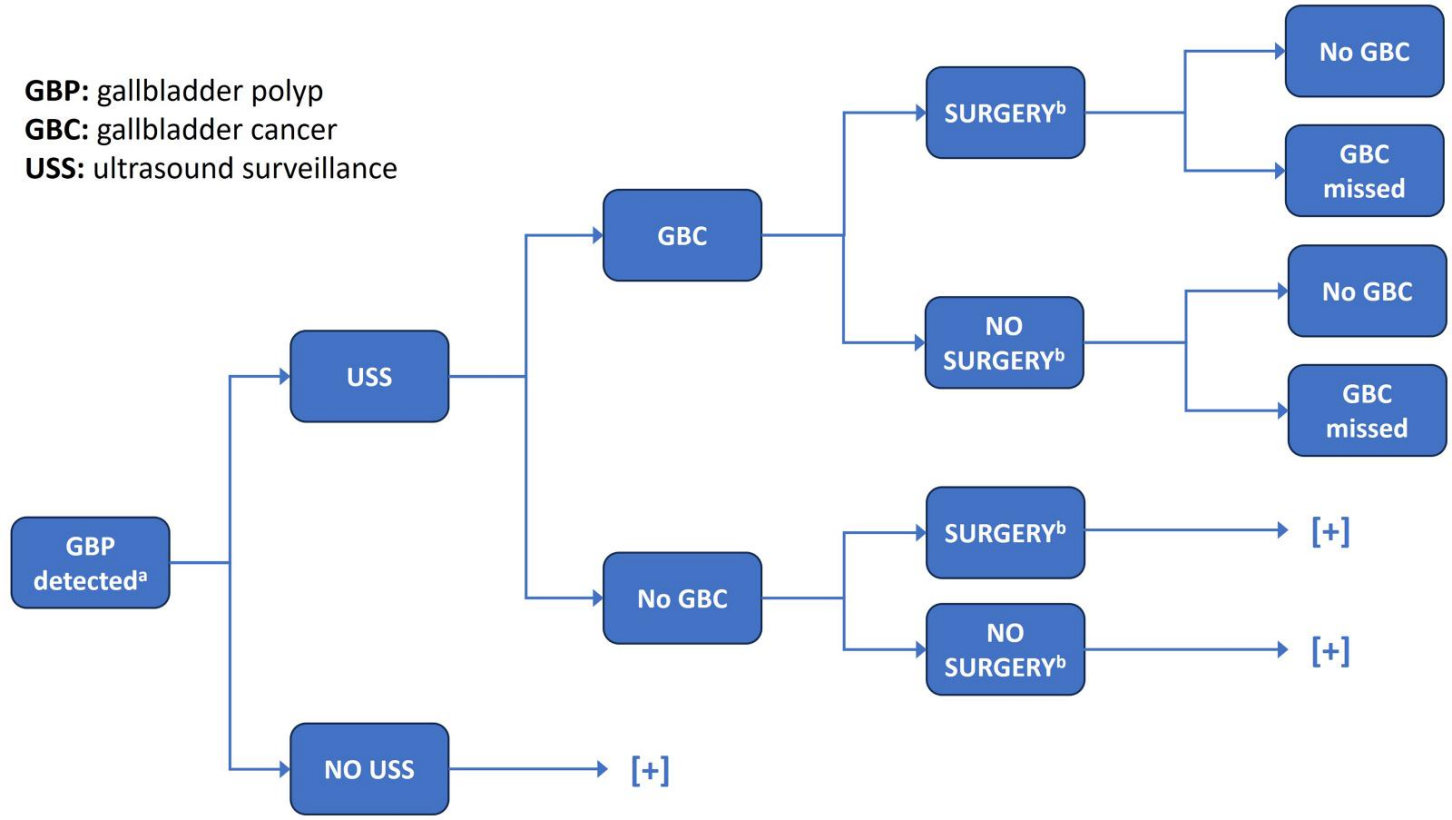
Model schematic. ^a^GBP detected at a size of either <6 mm or 6 mm to <10 mm (patient groups are modelled separately); ^b^Referral threshold for cholecystectomy applied of either an observed change in the GBP size of >2 mm, or an observed change in GBP size to >10 mm. [+] indicates decision tree branches are replications of the tree branches reported above. Abbreviations: GBC: gallbladder cancer; GBP: gallbladder polyp; USS: ultrasound surveillance.

We built a simple decision tree model in Microsoft Excel to replicate the expected management pathway of a patient enrolled in a GBP USS schedule (Figure 1). The objective of the analysis was to evaluate the cost-effectiveness of USS versus no USS in patients with GBPs <10 mm based on a hypothetical cohort of 1000 patients with an incidentally detected GBP. Model outcomes included expected numbers of TAUS and gallbladder surgeries (cholecystectomy), expected cases of GBC, expected costs of interventions (including TAUS and cholecystectomy), expected costs of GBC management, the net cost impact of USS versus no USS, and an estimate of the expected cost per GBC avoided. The model was built from the perspective of the UK NHS with a 2021/22 cost base in £sterling. The model timeframe was 2 years (in line with the current recommended duration of follow-up in this patient group).^2^

### Model development

During model development, we used systematic reviews and published evidence to construct a credible model and patient pathway.^2^^,^^7^^,^^12^ We also ran supplementary searches to identify additional GBP cohort studies and/or health economic evidence relevant to the cost-effectiveness of USS (see the Supplementary Material S1 for search terms). The patient pathway assumed that GBP patients would be referred for cholecystectomy according to the referral thresholds outlined in current guidelines, that is, an observed change in the GBP size of >2 mm, or an observed change in GBP size to >10 mm.^2^ We also evaluated the impact of a more conservative threshold in additional analysis (change in the size of GBP to >15 mm).

Given the nature of the pathway (repeated events), we considered alternate methodologies incorporating a Markov methodology; however, we did not retrieve data that facilitated this more granular approach. A key absence was data linking frequency of TAUS to the likelihood of the observation of a change in GBP size (the marker for surgical intervention). As such a more granular model was not possible. Similarly, we focused our methodology on cost-effectiveness as opposed to a more transferable assessment of cost-utility, as searches did not retrieve data that enabled a robust estimate of the impact that surveillance has on quality-adjusted life years (QALYs). The framework closely follows those pathways previously reported in UK observational studies which fit well with a decision tree methodology focusing on available and robust surveillance data.^10^^,^^11^

### Patient population

Our population comprised patients with a mean age of 50 years with incidentally detected GBPs <10 mm detected following routine TAUS for alternate conditions. To reflect the different underlying risks of GBC, we conducted separate analyses for patients with GBPs <6 mm and patients with GBPs 6–9 mm. This aligns with current guideline categorisation of GBC risk and GBP surveillance.^2^ Patients presenting with symptomatic GBPs or with GBPs >10 mm were not considered in this model. Patients who would not be eligible for cholecystectomy were also excluded from the model.

### Our analysis

Our analysis compared the recommended schedule of 3 TAUS scans over 2 years to no USS, in 2 risk groups (<6 mm and 6–9 mm) under **alternate thresholds** for cholecystectomy referral. Referral thresholds were based on published recommendations and included (1) an increase in GBP size of >2 mm (referral threshold 1) and (2) an increase in the total size of GBP to >10 mm (referral threshold 2) within 2 years of initial detection. These stratifications resulted in a total of 4 analysis groups (Table 1).

**Table 1.** Patient groups analysed in this study.

Management	Patient with GBPs < 10 mm on detection	
	GBP <6 mm	GBP 6–9 mm
USS follow-up	3 TAUS over 2 years	3 TAUS over 2 years
No USS follow-up	No routine TAUS	No routine TAUS
Surgical referral threshold	GBP growth >2 mm GBP grows to ≥10 mm	GBP growth >2 mm GBP grows to ≥10 mm

Abbreviations: GBP: gallbladder polyp; TAUS: transabdominal ultrasound; USS: ultrasound surveillance.

### Estimating clinical data

We did not conduct an independent systematic review for this exploratory analysis but referenced the literature described in the most recent guidelines and published systematic reviews.^2^^,^^12^ Model inputs were based on the findings of the large-scale cohort study referenced in the guidelines.^7^ This cohort study reported rates of GBC and the percentage of patients where a change in the size of GBP of >2 mm, or a total size change to 10 mm or more, was observed during USS, categorized by the initial size of GBP (<6 mm or 6–9 mm). No data that linked the frequency or intensity of TAUS to the likelihood of detecting changes in the size of GBP were available. The use of the cohort study data provided an objective marker for surgical referral and subsequent cholecystectomy allowing modelling of expected rates of cholecystectomy based on the initial size of GBP.

### Estimating the costs of management

The costs of USS and cholecystectomy were estimated from the 2021 NHS Schedule of Reference Costs assuming each TAUS conducted was 20 minutes or less according to standard practice, performed in an ambulatory setting, and that each cholecystectomy would be conducted in an elective outpatient setting.^13^ Weighted averages were estimated based on reported tariffs and activity numbers. There was limited literature estimating the direct costs of GBC management. The cost of GBC was estimated according to GBC management costs reported in a recent NICE clinical guideline and inflated to 2021/22 costs using HCHS indices^14^^,^^15^ Model inputs (including credible ranges) are reported in Table 2.

**Table 2.** Core model inputs.

Parameter		Base case	Upper^a^	Lower^b^	Reference
GBP < 6 mm		0.0013	0.0040	0	Szpakowski et al. (2020)^7^
GBP 6–9 mm		0.0087	0.0208	0	Szpakowski et al. (2020)^7^
GBC mortality		0.878	1	0.6146	NICE CG (2014)^14^
*P* change in size >2 mm^c^	GBP <6 mm	0.253	0.3289	0.1771	Szpakowski et al. (2020)^7^
	GBP 6–9 mm	0.207	0.2691	0.1449	Szpakowski et al. (2020)^7^
*P* change in size to >10 mm^d^	GBP <6 mm	0.05	0.065	0.035	Szpakowski et al. (2020)^7^
	GBP 6–9 mm	0.165	0.2145	0.1155	Szpakowski et al. (2020)^7^
Proportion FU before cholecystectomy^e^		0.5	0.25	0.75	ASSUMPTION
Drop out USS^f^		0		–	ASSUMPTION
GBC (£)		13 129	17 068	9190	NICE CG (2014)^14^
TAUS (£)		33	43	23	NHS Reference Costs (2019/20)^13^
Cholecystectomy (£)		2693	3500	1885	NHS Reference Costs (2019/20)^13^

a Inputs set to upper limit of plausible values.

b Inputs set to lower limit of plausible values.

c Referral threshold 1.

d Referral threshold 2.

e Baseline assumption is that surgery will occur half-way through follow-up, so patients meeting surgery thresholds incur 50% of USS resource.

f As there is no clear way to tie-in drop out to expected outcome, set to zero in our base case.

Abbreviations: FU: follow up; GBC: gallbladder cancer; GBP: gallbladder polyp; *P* = probability; TAUS: transabdominal ultrasound; USS: ultrasound surveillance.

### One-way sensitivity analyses and threshold analysis

We conducted extensive one-way sensitivity analyses (OWSA) around our base case analyses that considered referral threshold 2 (referral to cholecystectomy with a change in size to >10 mm). In the OWSAs, model inputs were varied between plausible upper and lower limits (based on 95% confidence intervals where possible) and reported visually in tornado diagrams. We also conducted threshold analysis to estimate the magnitude of GBC cost offset, in terms of the per case cost of GBC, that would be needed to effectively balance the additional costs of USS (i.e., the GBC per case cost at which USS delivery becomes cost neutral). Probabilistic sensitivity analysis was not conducted.

### Additional analysis

Additional analysis explored the impact that a more conservative threshold for surgical referral might have on expected costs. In this, we assumed that a clinically plausible adjustment to the threshold for cholecystectomy would be to consider a change in the size of GBP to >15 mm as the trigger for cholecystectomy referral (rather than the base case where we look at cholecystectomy on a change in the size of GBP to >10 mm).^16^ In this scenario, fewer patients meet the criteria for cholecystectomy. To quantify this in the model, we assumed that only a proportion of those GBPs that changed in size to >10 mm would change in size to >15 mm; in the absence of any evidence base, we assumed that proportion to be 40%. This analysis was conducted for both patient cohorts (GBPs <6 mm and GBPs 6–9 mm).

### Public and patient involvement

For the purpose of this exploratory analysis, we did not elicit public or patient involvement to validate the framework of the model, or the data and assumptions used to populate it. However, we recognize that this would be a valuable step in promoting any potential GBP pathway changes. No patients were involved in setting this specific research question and outcome measures, developing the study design or analysis plans, nor interpretation or reporting of results.

## Results

### Base case analysis

Base case results were estimated for each patient cohort and reported according to alternate thresholds for surgical referral, based on (1) a change in the size of >2 mm (referral threshold 1) and (2) a change in size to greater than 10 mm (referral threshold 2). For a cohort of 1000 patients enrolled in USS, where initially detected GBPs were <6 mm in size, we estimated a 2-year net cost impact of between £750 045 (referral threshold 1) and £213 441 (referral threshold 2), compared to the expected costs for 1000 patients not enrolled in USS. The total number of TAUS was estimated between 2621 and 2925, and expected numbers of cholecystectomies were estimated between 253 and 50. This balanced against 1.3 potential cases of GBC (at an estimated cost saving of £17 068) and 1.14 potential GBC-related deaths avoided. The incremental cost per GBC avoided was estimated at £576 958 (referral threshold 1) and £164 186 (referral threshold 2) (Table 3). Taking the example of the outputs for referral threshold 2 (GBP change in size to greater than 10 mm), we can infer that for each case of GBC avoided, a total of 2250 TAUS and 38 surgeries would be conducted.

**Table 3.** Analysis outputs, cohort size = 1000 patients with GBP <6 mm.

Referral threshold^a^	Change in size >2 mm^b^			Change in size to >10 mm^c^		
Model output	USS	No USS	Increment	USS	No USS	Increment
TAUS (n)	2621	0	2621	2925	0	2925
Cholecystectomy (n)	253	0	253	50	0	50
GBC (n)	0.00	1.30	−1.30	0.00	1.30	−1.30
GBC death (n)	0.00	1.14	−1.14	0.00	1.14	−1.14
TAUS (£)	85 900	0	85 900	95 882	0	95 882
GBC (£)	0	17 068	−17 068	0	17 068	−17 068
Cholecystectomy (£)	681 212	0	681 212	134 627	0	134 627
TOTAL (£)	767 113	17 068	750 045	230 509	17 068	213 441
Cost per GBC avoided (£)			576 958			164 186
Annual^d^ (£)	383 556	8534	375 023	115 254	8534	106 721

a Referral threshold linked directly to the change in size detected on US.

b Referral threshold 1.

c Referral threshold 2.

d Annual costs based on a 2-year total time horizon.

Abbreviations: GBC: gallbladder cancer; GBP: gallbladder polyp; TAUS: transabdominal ultrasound; USS: ultrasound surveillance.

For a cohort of 1000 patients enrolled in USS, where initially detected GBPs were 6–9 mm in size, we estimated the 2-year net cost impact of USS between £531 297 (referral threshold 1) and £420 275 (referral threshold 2). The total number of TAUS was estimated between 2690 and 2753, with expected numbers of cholecystectomies estimated between 207 and 165. This balanced against 8.7 potential cases of GBC avoided (at an estimated cost saving of £114 221) and 7.6 GBC-related deaths avoided. The incremental cost per GBC avoided was estimated at £61 069 (referral threshold 1) and £48 307 (referral threshold 2) (Table 4). Taking the example of the outputs for referral threshold 2 (GBP change in size to greater than 10 mm), we can infer that for each case of GBC avoided, a total of 316 TAUS and 19 surgeries would be conducted.

**Table 4.** Analysis outputs, cohort size = 1000 patients with GBP 6 mm to <10 mm.

Referral threshold^a^	Change in size >2 mm^b^			Change in size to >10 mm^c^		
Model output	USS	No USS	Increment	USS	No USS	Increment
TAUS (n)	2690	0	2690	2753	0	2753
Cholecystectomy (n)	207	0	207	165	0	165
GBC (n)	0.00	8.70	8.70	0.00	8.70	8.70
GBC mortality (n)	0.00	7.64	7.64	0.00	7.64	7.64
TAUS (£)	88 162	0	88 162	90 227	0	90 227
GBC (£)	0	114 221	−114 221	0	114 221	−114 221
Cholecystectomy (£)	557 356	0	557 356	444 269	0	444 269
TOTAL (£)	645 518	114 221	531 297	534 496	114 221	420 275
Cost per GBC avoided (£)			61 069			48 307
Annual^d^ (£)	322 759	57 111	265 648	267 248	57 111	210 138

a Referral threshold linked directly to the change in size detected on US.

b Referral threshold 1.

c Referral threshold 2.

d Annual costs based on a 2-year total time horizon.

Abbreviations: GBC: gallbladder cancer; GBP: gallbladder polyp; TAUS: transabdominal ultrasound; USS: ultrasound surveillance.

### One-way sensitivity analyses and threshold analysis

**Figure 2.**
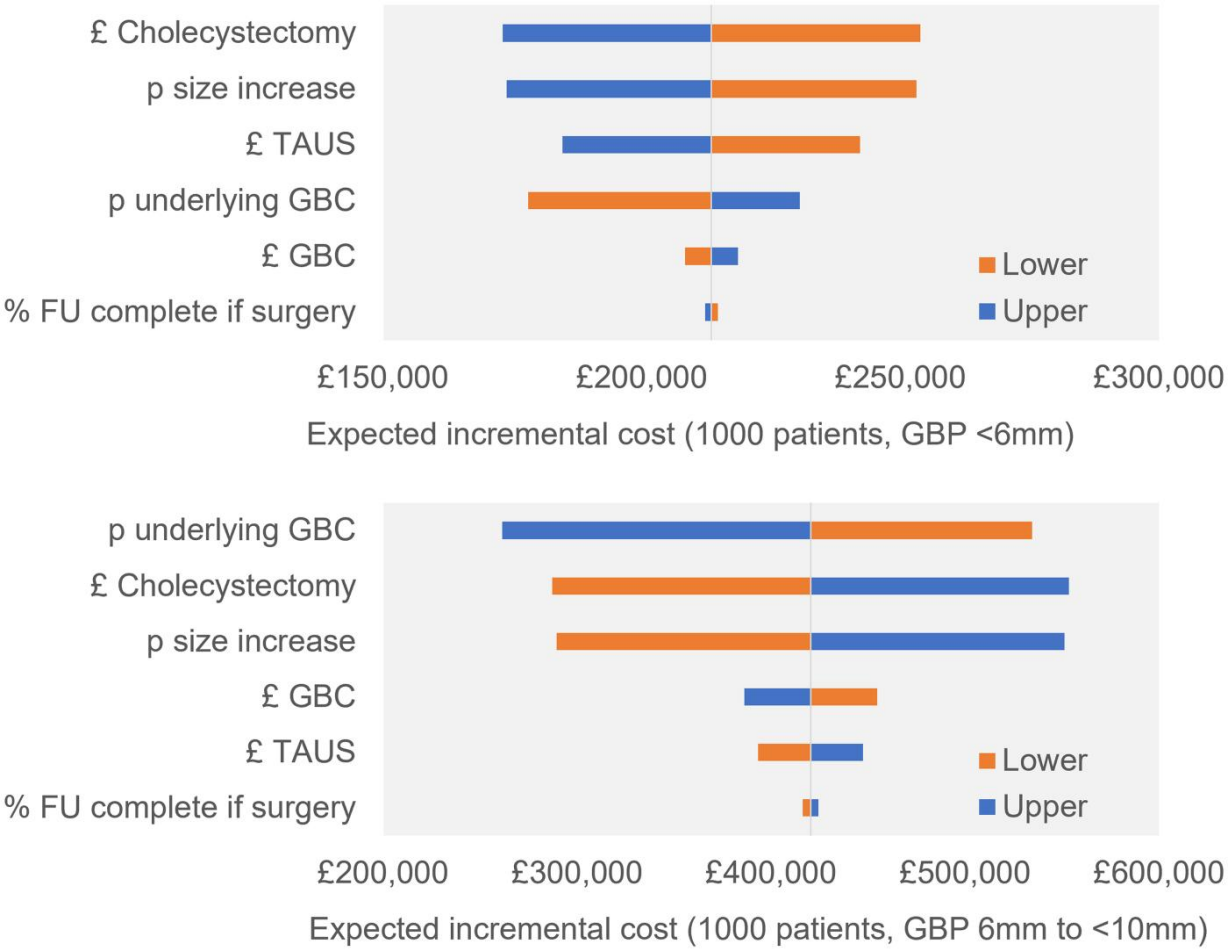
Tornado diagrams (surgery threshold based on polyp size increase to 10 mm). A tornado diagram is a graphical representation of uncertainty where the larger the line, the larger the impact of parameter uncertainty on the model outcome. Abbreviations: FU: follow-up; GBC: gallbladder cancer; p: probability; TAUS: transabdominal ultrasound.

OWSA results indicated that the finding of additional cost was robust but that the magnitude of additional cost varied considerably. Outputs are illustrated in Figure 2. Taking the example of the cohort of patients with GBPs <6 mm, the net 2-year cost impact varied from an additional £173 053 (£173 per patient) when the lower cost limit of cholecystectomy was applied through to an additional £253 830 (£254 per patient) when the upper limit to the cost of cholecystectomy was applied. For the cohort of patients with GBPs 6–9 mm, the net 2-year cost impact varied from an additional £261 416 (£261 per patient) when the upper rate of GBC was applied, through to an additional £553 556 (£554 per patient) when the upper limit to the cost of cholecystectomy was applied. In threshold analysis, for the patient cohort with GBPs <6 mm, the case cost of GBC required to offset the additional costs of USS was estimated at £590 100 (referral threshold 1) and £177 300 (referral threshold 2). For the patient cohort with GBPs 6–9 mm, these same metrics were estimated at £74 200 and £61 400.

### Additional analysis

Outputs of the additional analysis are reported in Table 5. For the cohort of patients where initially detected GBPs were <6 mm, a hypothetical increase in the threshold for surgical referral to a change in the size of GBP >15 mm resulted in 2-year estimated net costs of £134 140 alongside 2970 TAUS and a total of 20 additional cholecystectomies, that is, an expected total of 2285 TAUS and 15 surgeries for every expected case of GBC. The incremental cost per GBC avoided was estimated at £103 185 (versus £164 186 at the original referral threshold). For the cohort of patients where initially detected GBPs were 6–9 mm, a hypothetical increase in the threshold for surgical referral resulted in 2-year estimated net costs of £158 582 alongside 2900 USS-related ultrasounds and a total of 66 additional surgeries, that is, an expected total of 333 TAUS and 8 surgeries for every expected case of GBC. The incremental cost per GBC avoided was estimated at £18 228 (versus £48 307 at the original referral threshold).

**Table 5.** Analysis outputs, cohort size = 1000 patients, exploratory threshold for cholecystectomy (size change to >15 mm).

GBP initial size	GBP <6 mm			GBP 6 mm to <10 mm		
Model output	USS	No USS	Increment	USS	No USS	Increment
TAUS (n)	2970	0	2970	2901	0	2901
Cholecystectomy (n)	20	0	20	66	0	66
GBC (n)	0.00	1.30	−1.30	0.00	8.70	−8.70
GBC mortality (n)	0.00	1.14	−1.14	0.00	7.64	−7.64
TAUS (£)	97 357	0	97 357	95 095	0	95 095
GBC (£)	0	17 068	−17 068	0	114 221	−114 221
Cholecystectomy (£)	53 851	0	53 851	177 708	0	177 708
TOTAL (£)	151 208	17 068	134 140	272 803	114 221	158 582
Cost per GBC avoided (£)			103 185			18 228
Annual^a^ (£)	75 604	8534	67 070	136 401	57 111	79 291

a Annual costs based on a 2-year total time horizon.

Abbreviations: GBC: gallbladder cancer; GBP: gallbladder polyp; TAUS: transabdominal ultrasound; USS: ultrasound surveillance.

## Discussion

Our cost-effectiveness analysis found that compared to no follow-up, USS and associated referral for cholecystectomy resulted in increased costs across all scenarios, with considerable 2-year net costs in patients with GBPs of <6 mm and 6–9 mm, depending on the surgical referral threshold. These costs were a function of high numbers of additional surgeries balanced against marginal numbers of GBC avoided. OWSA indicated that although the magnitude of cost varied, the finding of increased cost with USS versus no USS was robust to plausible input changes under current model assumptions (no OWSA resulted in a shift to cost saving). These results support recent guideline updates no longer recommending USS in patients with GBPs <6 mm but question the rationale for continued recommendation of USS in patients with GBPs of 6–9 mm in the UK NHS.

The core aim of our analysis was to determine whether it was possible to estimate the cost-effectiveness of USS in patients with GBPs <10 mm based on a robust and transparent dataset of model inputs that would be broadly applicable to the NHS healthcare setting and broadly generalizable to a UK population. Our analysis suggests that if thresholds for referral for cholecystectomy were employed rigidly within the USS schedule, a high number of cholecystectomies would be performed within the UK NHS setting. If we extrapolate our numbers, to estimate outcomes per 100 000 population, we might expect 625 patients with incidental GBPs <10 mm (based on a 2.5% prevalence and 25% of GBPs being <10 mm) and, based on our current model assumptions, between 89 and 135 surgeries, dependent on the surgical referral threshold applied (assuming 20% of our cohort have GBPs <6 mm).^12^ This would compare against 4.5 potential cases of GBC in the same cohort, suggesting that in over 95% of the surgeries, we would not expect underlying GBC. A formal assessment of cost-effectiveness was not possible given the availability and differentiation of currently available data.

Our analysis explored the resource and cost impact of USS with specific focus on the impact that different thresholds for surgical referral would have on expected rates of cholecystectomy. As such, our findings may not be directly comparable to recently reported UK cost studies. These studies both estimated substantial institution-based savings through USS follow-up of GBPs <10 mm of between £132 000 and £167 000.^10^^,^^11^ In our analyses, the costs associated with TAUS and subsequent cholecystectomy consistently outweighed the potential cost offsets associated with avoiding future GBC. Differences in methodological approach help to explain these differences. In our analysis, we explored the impact of the strict application of the surgical referral thresholds cited in current guidelines on NHS resources, with rates of cholecystectomy estimated based on the expected outcomes of USS (observed changes in GBP size), whereas previous studies calculated total USS costs based on institution-reported numbers of surgery. While the rates of cholecystectomy for those patients enrolled in formal USS were similar across our studies (the UK studies reported 16% and 28% compared to our outcome-based estimates of between 17% and 21%), outcomes for patients not enrolled in formal USS were also included in the published studies, which might act to dilute the costs associated with USS delivery. In addition, the cost of GBC was estimated differently. The UK cost studies both applied the same GBC cost in their analyses, derived in the first of the 2 studies by dividing the total cost of UK cancer care (reported at £18.3 billion) by the expected numbers of incident cases of cancer (reported at 309 500).^10^ This resulted in their use of £60 000 cost per GBC case avoided. We based the cost of GBC management in our analysis (£13 129) on the costs of GBC previously reported in a UK NICE clinical guideline for management of gallbladder diseases,^14^ under the assumption that these costs may be more consistent with the NHS management costs expected in advanced GBC. Further research to better define the cost of the management of GBC would be a key step toward formal assessment of the cost-effectiveness of USS monitoring in a UK NHS setting.

There are limitations to this study. We attempted to produce an estimate of cholecystectomy rates based on objectively measured outcomes of the USS scheme, to predict the potential impact of guideline compliance on numbers of cholecystectomies at a given department. However, we acknowledge that the rates of cholecystectomy are, in practice, unlikely to match the rates of referrable GBPs (eg, variations in the application of referral thresholds, variations in the numbers of patients proceeding to cholecystectomy) and the true numbers of cholecystectomies undertaken may be lower than our estimated rates. This would mean that the costs allocated to USS may be an over-estimate (although consistent with our aim to estimate the potential cost and resource impact of adherence to guideline recommendations). The model followed other published analyses in assuming that any potential GBC would be captured by change in the size (i.e., surgical referral) pathway. We assumed that while not all GBPs that change in the size would lead to GBC, all GBP-related GBC would follow a change in the size of GBP (ie, we would not expect GBP-related GBC to develop in patients whose GBPs remain stable and/or do not meet the stated thresholds for surgical referral). While consistent with other published analyses,^10^^,^^11^ the potential error concerning numbers of over-estimated GBC avoided needs further exploration. We assumed that the underlying rates of GBC applied in our analysis are reflective of expected rates of GBC in a UK population, and this may not be accurate. Detailed UK-specific data were not available, but the underlying rates of GBC being 0.13% (95% confidence interval 0% to 0.4%) in GBPs <6 mm and 0.87% in GBPs between 6 and 9 mm (95% confidence interval 0% to 2.1%) are in line with meta-analyses that estimated the cumulative malignant risk of GBC in polyps measuring 5 mm and 9 mm at 0.14% (99% credible range 0.08%–0.26%) and 0.51% (99% credible range 0.26%–0.97%), respectively.^12^ Additional capture of UK-specific data could be warranted. Finally, available GBP data reviewed in this study emphasized uncertainties in the GBP evidence base. Based on inputs identified for the analysis, the link between GBPs below the 10 mm threshold and subsequent risk of GBC does not appear well established (credible limits around our base-case inputs include zero) which is problematic for USS and warrants additional research.

Despite these limitations, our analysis used the best available data to present a transparent platform for evaluating the cost-effectiveness of USS within the UK NHS. Overall, our analyses suggested that, based on currently available data, wide-scale USS follow-up in patients with GBPs less than 10 mm is unlikely to be a cost-effective use of NHS resources. While small clinical gains would be made in terms of potential cases of GBC avoided, these gains need to be balanced against the weight of resource required to deliver the scheme, in particular the number of cholecystectomies that could be expected if the thresholds suggested in current guidelines were strictly adhered to. However, it is challenging to formally address the question of cost-effectiveness without UK-specific data and without a better understanding of the link between frequency, timing, and the observed outcomes of TAUS. UK-based audits of USS that capture these metrics would allow for more sophisticated analysis and confirm the patient impact and cost implications of USS in this GBP cohort.

## Conclusion

This study suggests that USS of patients with GBPs less than 10 mm may not be a cost-effective use of limited resources. However, the current evidence is of low quality. Based on our analyses, current USS in these patients should be questioned. We have identified an important gap in the literature and recommend that further real-world-evidence to better define both patient and economic outcomes is needed. This research should include consideration of the practical sustainability of high rates of TAUS and cholecystectomy within the context of current NHS budget and resource constraints.

## Supplementary Material

tqaf024_Supplementary_Data
