# Supplementary material for: Cost-effectiveness of trans-abdominal ultrasound for gallbladder cancer surveillance in patients with gallbladder polyps less than 10 mm in the United Kingdom
Source: Br J Radiol. 2025 Feb 5;98(1169):693–700. doi: 10.1093/bjr/tqaf024 (PMC12012355; doi:10.1093/bjr/tqaf024)
Supplement: tqaf024_Supplementary_Data [file tqaf024_supplementary_data.docx]

SUPPLEMENTARY MATERIAL

Search Terms

Supplementary searches were conducted in EMBASE using the following terms:

gallbladder:ab,ti AND polyp:ab,ti AND malignancy:ab,ti AND [2011-2022]/py

gallbladder:ab,ti AND polyp:ab,ti AND cost:ab,ti AND [2011-2022]/py

((gallbladder NEXT/2 cancer):ab,ti) AND cost:ti AND [2011-2022]/py
